# Betahistine Exerts a Dose-Dependent Effect on Cochlear Stria Vascularis Blood Flow in Guinea Pigs *In Vivo*

**DOI:** 10.1371/journal.pone.0039086

**Published:** 2012-06-20

**Authors:** Fritz Ihler, Mattis Bertlich, Kariem Sharaf, Sebastian Strieth, Michael Strupp, Martin Canis

**Affiliations:** 1 Department of Otorhinolaryngology, Head and Neck Surgery, Goettingen University Medical School, Göttingen, Germany; 2 Integrated Center for Research and Treatment of Vertigo, Balance and Ocular Motor Disorders, University of Munich Hospital, Munich, Germany; 3 Walter Brendel Centre of Experimental Medicine, University of Munich Hospital, Munich, Germany; 4 Department of Otorhinolaryngology, Head & Neck Surgery, J. W. Goethe-University-Medical School, Frankfurt/Main, Germany; 5 Department of Neurology, University of Munich Hospital, Munich, Germany; University of Minnesota Medical School, United States of America

## Abstract

**Objective:**

Betahistine is a histamine H_1_-receptor agonist and H_3_-receptor antagonist that is administered to treat Menière’s disease. Despite widespread use, its pharmacological mode of action has not been entirely elucidated. This study investigated the effect of betahistine on guinea pigs at dosages corresponding to clinically used doses for cochlear microcirculation.

**Methods:**

Thirty healthy Dunkin-Hartley guinea pigs were randomly assigned to five groups to receive betahistine dihydrochloride in a dose of 1,000 mg/kg b. w. (milligram per kilogram body weight), 0.100 mg/kg b. w., 0.010 mg/kg b. w., 0.001 mg/kg b. w. in NaCl 0.9% or NaCl 0.9% alone as placebo. Cochlear blood flow and mean arterial pressure were continuously monitored by intravital fluorescence microscopy and invasive blood pressure measurements 3 minutes before and 15 minutes after administration of betahistine.

**Results:**

When betahistine was administered in a dose of 1.000 mg/kg b. w. cochlear blood flow was increased to a peak value of 1.340 arbitrary units (SD: 0.246; range: 0.933–1.546 arb. units) compared to baseline (p<0.05; Two Way Repeated Measures ANOVA/Bonferroni t-test). The lowest dosage of 0.001 mg/kg b. w. betahistine or NaCl 0.9% had the same effect as placebo. Nonlinear regression revealed that there was a sigmoid correlation between increase in blood flow and dosages.

**Conclusions:**

Betahistine has a dose-dependent effect on the increase of blood flow in cochlear capillaries. The effects of the dosage range of betahistine on cochlear microcirculation corresponded well to clinically used single dosages to treat Menière’s disease. Our data suggest that the improved effects of higher doses of betahistine in the treatment of Menière’s disease might be due to a corresponding increase of cochlear blood flow.

## Introduction

In 1861 Prosper Menière (1799–1862) described a typical combination of symptoms (hearing loss, tinnitus, and attacks of vertigo) [1]. Only years later Jean-Martin Charcot (1825–1893) named the disorder in his honor “Menière’s triad”. Menière ascribed the condition to a lesion in the semicircular canals, thereby challenging the then prevailing opinion that vertigo was a condition caused exclusively by pathologies of the central nervous system [2]. Despite considerable research in this field since the time of Menière, the malady still causes a high burden of disease with an estimated lifetime prevalence of 0.51% [3]. Moreover, there is still no consensus on its pathophysiology or treatment.

A wide range of treatment strategies is now applied; however, reliable evidence of their efficacy is scarce for most of them, and their side effects are severe. They include diuretics [4], intratympanic application of gentamicin [5], steroids [6] or endolymphatic sac surgery [7].

Another option for long-term treatment is the oral administration of betahistine, a structural analog of histamine that has strong antagonistic effects on histamine H_3_ receptors and weak agonistic effects on histamine H_1_ receptors [8], [9]. Its mechanism of action is not yet fully understood. Clinical trials and meta-analyses suggest that betahistine might have a beneficial effect on patients with Menièrès disease, especially on the frequency of attacks [10], [11], [12]. An open trial found that this effect might be dose dependent: a dosage of 48 mg tid (three times a day, i.e. 144 mg per day), was shown to be more effective than 16 to 24 mg tid [13]. More recently even higher dosages of 240 to 480 mg per day had a significant effect on the frequency of attacks [14]. Nevertheless, there is still a lack of well-conducted, controlled, double-blind randomized prospective clinical trials on the application of betahistine in Menière’s disease.

To date, several animal studies have shown that betahistine promotes cochlear blood flow in vivo [15], [16], [17], [18], [19]. The dosage of betahistine administered intravenously in these studies ranged from 0.1 to 10.0 mg/kg b. w. None of these studies found a dose-response curve. Corresponding doses for clinical use range from about 0.05 mg/kg b. w. to 0.6 mg/kg b. w. orally [12], [13], [14]. Due to the drug’s delayed bioavailability during enteral resorption and strong first-pass effect, the resulting serum concentration after oral betahistine is estimated to be much lower than from intravenous application. Thus, much higher doses need to be given orally to achieve comparable plasma concentrations. In the current study, we examined i. v. dosages calculated to correspond to oral dosages given to patients. They ranged from 0.001 to 1,000 mg betahistine/kg b. w.

The aim of this study was to evaluate the effects of clinically relevant dosages of betahistine on the microcirculation of cochlear lateral wall vessels in order to elucidate the drug’s possible mode of action in Menière’s disease.

## Materials and Methods

### Ethics Statement

All experiments were performed according to Bavarian state regulations for animal experimentation and were approved on 18/04/2011 by the responsible authorities, the District Government of Upper Bavaria (Regierung von Oberbayern Munich, Germany; animal license no.: 55.2-1-54-2532-131-10).

### Animals

Thirty healthy female Dunkin-Hartley guinea pigs (250–400 g) obtained from Charles River Laboratories (Sulzfeld, Germany) were included in the study. The animals were first sedated for 10 minutes with 1 l O_2_/min, 0.5 l N_2_H/min with 2 Vol-% halothane in a small plastic chamber before the induction of anaesthesia. They then received an intraperitoneal injection of 50.0 mg/kg b. w. ketamine and 5.0 mg/kg b. w. xylazine. Anaesthesia was continued throughout the experiments with intraperitoneal injections of 25.0 mg/kg b. w. ketamine and 2.5 mg/kg b. w. xylazine given every 30 min.

The preparative surgery in the experiments lasted on average about 1½ h and the measurements 18 minutes. Following the experiments, the animals were euthanized.

### Surgical Preparation and Intravital Imaging

Surgical preparation and intravital microscopy for measuring microcirculation parameters were performed as described elsewhere [20]. Heart rate and oxygen saturation were continuously monitored using pulsoxymetry. A fiberoptic pressure transducer was placed in the left femoral artery for blood pressure measurement (see below) and in the left jugular vein for intravenous injections. Through a post auricular incision, the right auditory bulla was opened, the cochlea itself was exposed, and a rectangular window (0.2×0.3 mm) was incised at the second turn.

Subsequently, intravital microscopy allowed direct observation of cochlear lateral wall vessels and the measurement of red blood cell velocity v (in µm/s) and vessel diameter (in µm). Fluorescein isothiocyanate (FITC)-labeled dextran (molecular weight 500,000; 0.05–0.1 ml of a 5% solution in0.9% NaCl; Sigma, Deisenhofen, Germany) was injected intravenously as a plasma marker to differentiate plasma from red blood cells, which do not absorb the dye. Selective observation of FITC-labeled plasma was performed using epi-illumination with a 100 W mercury lamp attached to a specific fluorescence filter block (excitation 450–490 nm, emission 515 nm), mounted on a modified Zeiss microscope (AxiotechVario; Zeiss, Goettingen, Germany). Images were acquired by video camera (C2400-08; Hamamatsu, Herrsching, Germany) and recorded on digital video tape (Sony DVCAM DSV 45 P; Sony, Cologne, Germany) for subsequent analysis. Red blood cell velocity v [µm/s] and vessel diameter d [µm] were measured off-line using an image analysis system (Cap Image; Zeintl, Heidelberg, Germany), described in detail elsewhere [21]. For the current study cochlear blood flow was assessed at a given point of time in three independent vessels chosen for analysis and was averaged. A line-shift diagram for each vessel allowed calculation of red blood cell velocity v for 7 s [µm/s], while the average of three independent measurements provided vessel diameter d [µm]. To quantify blood volume per time flowing through chosen vessels, cochlear blood flow q (in pl/s) was calculated from these parameters by the formula described by Baker and Wayland [22]: q = (v/1.6) * (d/2)^2^ * π. To correct for differences due to the varying size of vessels within the cochlear window, cochlear blood flow is reported as an arbitrary unit (arb. unit), reflecting relative change from basal blood flow in each vessel.

### Systemic Measurement of Blood Pressure

Mean arterial pressure was assessed by a fiberoptic pressure catheter system (Samba Preclin, Samba Sensors AB, VästraFrölunda, Sweden) [23]. A pressure transducer with a diameter of 0.42 mm (Samba Preclin 420 transducer, 5 cm bare fiber length) was inserted during microsurgery into the left femoral artery under anaesthesia. Data acquisition took place by a Samba 201 Control Unit; the Samba 200 Software Package was then used for analysis. Blood pressure was measured in millimetres of mercury (mmHg). To correct for values between different animals due to measurement inaccuracy and individual factors, mean arterial pressure is reported as an arbitrary unit (arb. unit), reflecting relative change from basal pressure.

### Treatment Protocol

Six animals were randomly assigned to 5 groups for treatment with 4 different concentrations of betahistine dihydrochloride (B4638, Sigma Aldrich, St. Louis, MO, USA) and to one control group that received NaCl 0.9% as placebo. All groups underwent identical surgery as described above. Baseline measurements of cochlear blood flow and mean arterial pressure were initiated. After 3 minutes either placebo or a dedicated concentration of betahistine was infused for 2 minutes. Cochlear blood flow and mean arterial pressure were continuously monitored from the onset of baseline measurements for a total of 18 minutes.

### Calculation of Corresponding Dosages for Oral Medication

In order to extrapolate our conclusions to the clinical situation, in which betahistine is administered orally, we calculated the serum levels of betahistine achieved after intravenous administration to guinea pigs and compared them to the known maximum plasma concentrations of 2-pyridylacetic acid achieved after oral administration to humans [24], [25]. 2-Pyridylacetic acid is a major metabolite of betahistine that results after the first-pass effect. It is commonly used as a measure of the betahistine pharmacokinetics, since there is currently no adequate means for quantifying betahistine serum levels directly. Chen et. al. reported that 339.4 ng/ml was the maximum plasma concentration (*C*_max_) after intake of a single dose of 24 mg betahistine [25]. An ideal guinea pig with 0.250 kg b. w. and about 12 ml blood plasma was estimated to reach this plasma concentration immediately after intravenous infusion of 0.0163 mg/kg b. w.

### Statistical Analysis

Statistical analyses were carried out by SigmaPlot 2004 for Windows Version 9.01 (Systat Software, Chicago, IL, USA). To detect significant differences, Two Way Repeated Measures ANOVA (with time set as repeated factor) was used to compare measurements at each time point from the treatment groups to the same time point of the placebo group.

Correction for multiple testing was performed for testing over multiple groups and multiple consecutive timepoints by Bonferroni t-test. A P-value of α <0.05 was considered to be statistically significant.

Nonlinear regression was used to fit a curve using selected data points. Results of nonlinear regression are reported as predicted residual sums of squares statistic (PRESS) and Kolmogorov-Smirnoff (K–S) Statistic D_n_. The fit was assessed by Adjusted R-squared 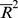.

## Results

### The Effect of Betahistine on Cochlear Microcirculation

**Figure 1:**
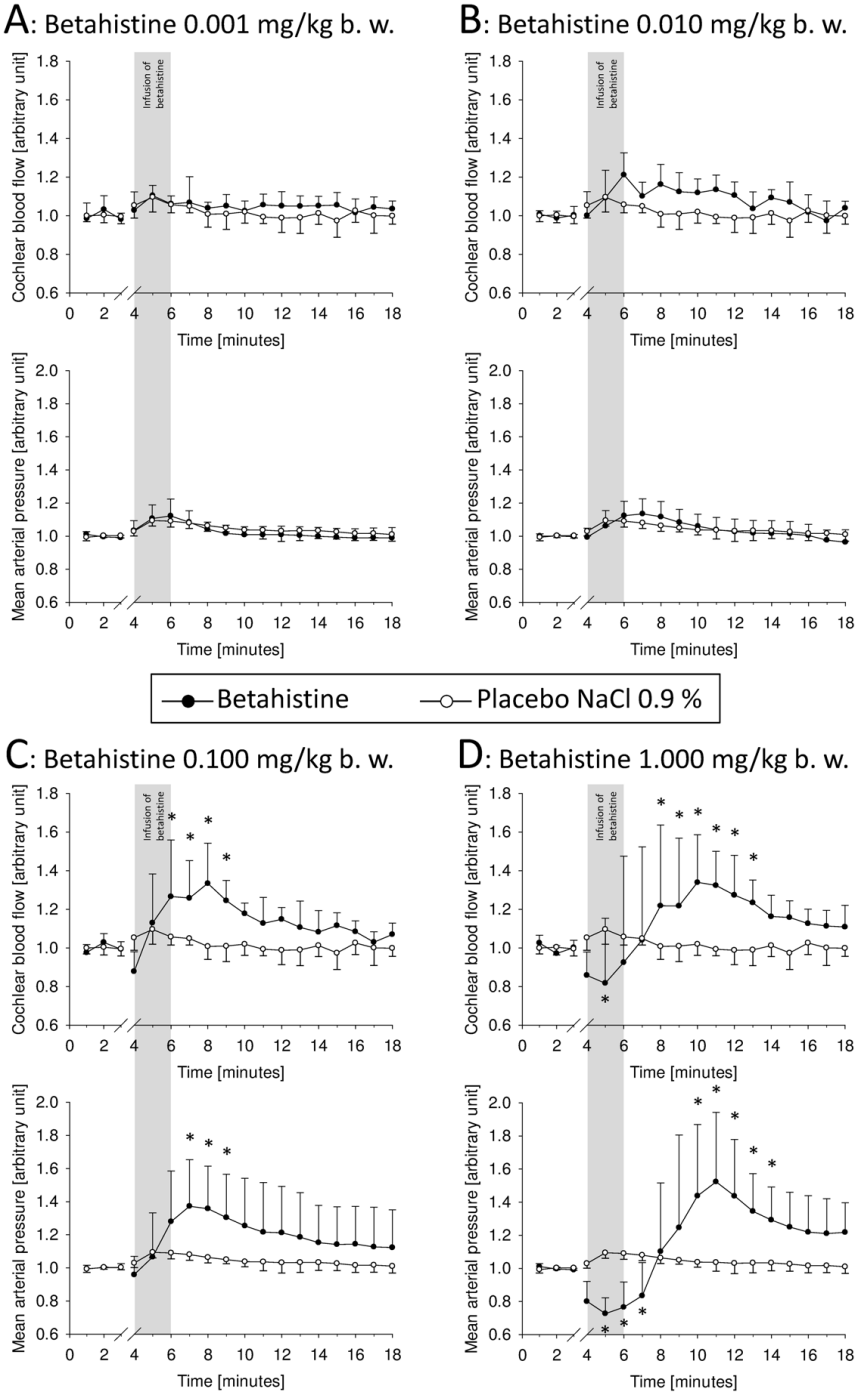
Cochlear blood flow and mean arterial blood flow over time before and after infusion of betahistine. A: Betahistine 0.001 mg/kg b. w.; B: Betahistine 0.010 mg/kg b. w.; C: Betahistine 0.100 mg/kg b. w.; D: Betahistine 1.000 mg/kg b. w.; arbitrary units, mean ± SD, *: p<0.05.

Administration of 0.001 and 0.010 mg betahistine/kg b. w. did not lead to significant changes in blood flow (Figure 1A, Figure 1B).

The treatment group receiving 0.100 mg betahistine/kg b. w. showed elevated cochlear perfusion after several minutes. Compared to placebo values, perfusion was significantly augmented during minutes 6 to 9 (p<0.05; Two Way RM ANOVA/Bonferroni t-test). Subsequently there was a further decrease in cochlear blood flow, but no more significant differences were observed (Figure 1C).

The effect on cochlear microcirculation was most pronounced in the treatment group receiving betahistine in a dosage of 1.000 mg/kg b. w. Here, cochlear blood flow showed a steep increase up to a mean peak value of 1.340 arb. units (SD: 0.246; range: 0.933–1.546 arb. units) at minute 10. This was significantly different from placebo values from minute 8 on (p<0.05; Two Way RM ANOVA/Bonferroni t-test). After reaching a peak value, cochlear blood flow decreased, and from minute 14 on there was no longer a significant difference from placebo values (Figure 1D).

Surprisingly, the administration of higher dosages of betahistine led to an initial drop in cochlear blood flow. This was less distinct when 0.10 mg/kg b. w. betahistine was dispensed (Figure 1C). A marked drop in cochlear blood flow took place almost immediately during infusion of 1.000 mg/kg b. w. betahistine (Figure 1D). At minute 5, cochlear blood flow was significantly reduced to 0.818 arb. units (SD: 0.336; range: 0.600–1.488 arb. units; p = 0.002; Two Way RM ANOVA/Bonferroni t-test).

### Effect of Betahistine on Systemic Blood Pressure

A basal mean arterial pressure (MAP) of 35.96 mmHg was the average of all animals tested (n = 30; SD: 7.76; range: 17.42–56.00 mmHg). After treatment with betahistine, dose-dependent changes were noted in MAP when compared to placebo.

Treatment groups receiving 0.01 and 0.001 mg betahistine/kg b. w. showed no noticeably significant differences in systemic blood pressure from the placebo group at any time point (Figures 1A and 1B).

A dosage of 0.100 mg/kg b. w. betahistine led to a MAP that peaked at 42.97 mmHg (SD: 6.66; range: 38.07–51.59 mmHg) or 1.373 from basal MAP at minute 7 (Figure 1C). This finding and the values at minute 8 and 9 were significantly different from placebo (p<0.05; Two Way RM ANOVA/Bonferroni t-test).

In the group treated with the highest dose of betahistine (1.000 mg/kg b. w.), a sharp increase in MAP was observed which reached a maximum of 64.10 mmHg (SD: 13.20; range: 47.76–78.56 mmHg) at minute 11. This is equivalent to a relative change to 1.523 arb. units compared to a mean basal MAP of this group. Compared to the control group the values from minute 10 to 14 were statistically significant (p<0.05; Two Way RM ANOVA/Bonferroni t-test). Thereafter the MAP decreased until the end of the recording period (Figure 1D).

Like cochlear blood flow, the MAP also significantly decreased after intravenous administration of 1.000 mg/kg b. w. betahistine from minute 4 on; 33.09 mmHg was the minimal turning point at minute 5 (SD: 5.21; range: 26.92–37.92 mmHg; Figure 1D). This corresponds to a fraction of 0.727 from mean basal MAP of this group. The difference from the placebo group was statistically significant from minute 5 to 7 (p<0.05; Two Way RM ANOVA/Bonferroni t-test).

### Regression Analysis and Calculated Equivalent Oral Dosage in Humans

A sigmoid curve was fitted by nonlinear regression of the peak values of cochlear blood flow, thereby suggesting a saturation effect for doses greater than 0.1 mg/kg b. w. (PRESS = 0.0059; D_n_ = 0.2231 with significance level = 0.9726). The fit was assessed by Adjusted R-squared with 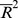 = 0.9996 (SD = 0.0025), implying a good approximation of the given variables by the fitted curve.

**Figure 2:**
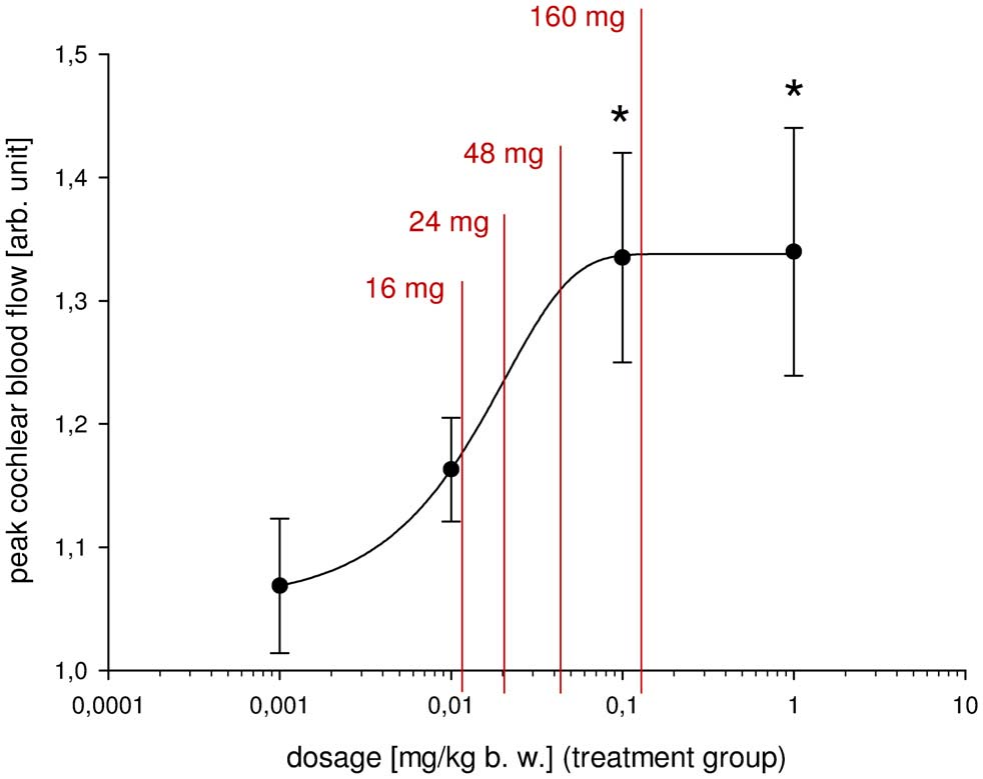
Peak values of cochlear blood flow with curve fitted by nonlinear regression and calculated corresponding oral dosage. Four treatment groups as described in the text; peak mean ± SD; *: p<0.05.

The corresponding dosage for oral administration to humans was calculated as described above: a dose of 16 mg betahistine orally corresponded to intravenous administration of 0.0109 mg/kg b. w., 24 mg to 0.0163 mg/kg b. w., 48 mg to 0.0259 mg/kg b. w. and 160 mg to 0.1086 mg/kg b. w., respectively. Figure 2 shows that these dosages lie within a range in which a steep dose-dependent increase of cochlear blood flow was found after increased administration of betahistine.

## Discussion

The major findings of this study were as follows: betahistine has a dose-dependent augmentative effect on both cochlear blood flow and systemic blood pressure. Several groups have already examined the effects of intravenous betahistine on inner ear microcirculation [15], [16], [17], [18], [19] and reported an increase of cochlear or vestibular blood flow. Our data are in line with the previously reported results, but with three major differences. First, the earlier experiments generally applied laser Doppler flowmetry to quantify cochlear blood flow; however, it does not readily allow conclusions to be drawn on blood flow changes in the capillaries of the cochlear lateral wall. Second, the dosages administered in animal studies before were not the doses used in the treatment of Menière’s disease: they were much higher. Third, none of the earlier studies found a sigmoid dose-response curve.

Laser Doppler flowmetry has been used to quantify cochlear microcirculation since the 1980s [26], [27], [28], [29]. An alternative technique, which is – as discussed below - more elaborate, is intravital flourescence microscopy, which was applied in this study. It relies on direct observation of cochlear lateral wall vessels [20], [30], [31], [32], [33] and was more appropriate for the present investigation, since intravital flourescence microscopy allows direct measurement of blood flow exclusively in capillaries of cochlear stria vascularis. In contrast laser Doppler flowmetry, which also gives a good estimation of blood flow in the cochlea, is in fact an average signal of any vessel beneath the Doppler probe, including among others the spiral modiolar artery and vessels of the bony capsule that belong to middle ear circulation [31], [34].

Our current understanding of the pathophysiology of Menière’s disease is that a relative overproduction of endolymph occurs in the membranous labyrinth of the inner ear with periodical rupture or leakage of Reissner’s membrane [35], [36], [37]. The mode of action of betahistine in Menière’s disease is that it probably increases inner ear blood flow and thereby reduces endolymphatic hydrops by shifting the balance of production and re-absorption of endolymph toward absorption. Since the capillaries of the cochlear lateral wall are the main location of cochlear metabolism, the present study focused solely on these vessels, thereby contributing to the understanding of blood flow regulation by betahistine.

To date, betahistine has been administered intravenously in every animal model used to investigate the effect of betahistine on inner ear microcirculation [15], [16], [17], [18], [19]. Clinically it is only administered orally. This leads to different pharmacokinetics, since enteral absorption is followed by a considerably decelerated bioavailability. Additionally, a strong first-pass effect of betahistine has to be taken into account. To be able to compare animal studies with clinical studies using oral administration, we calculated the plasma concentration of betahistine achieved by intravenous injection of guinea pigs and designed the present study according to the results. The data presented here taken together with the pharmacokinetic of betahistine in human test persons [24], [25] leads to the conclusion that a further increase in cochlear perfusion in patients suffering from Menière’s disease might be achieved by doses higher than currently applied. While this calculation allows only a very rough estimation of plasma concentrations, we believe that it is much more instructive than directly comparing dosage administered via oral intake versus intravenous administration as in earlier experimental concepts. In the past, dosages of 4 mg four times a day and up to 24 mg three times a day were considered effective [12]. Our data suggest that higher doses might have an even more pronounced effect on cochlear perfusion. Two clinical trials support our view; they found that the dosages of 48 mg tid are more effective than 16 or 24 mg tid [13] and even higher dosages of 160 mg tid [14] are even more effective. Additionally, time T_max_ from oral administration to peak concentration has been shown to be 0.79 h (SD: 0.32 h) to 0.98 (SD: 0.47) in healthy volunteers [24], [25]. Assuming that Menière’s disease can be treated by betahistine through increasing cochlear perfusion and thereby reducing a causative endolymphatic hydrops, we conclude that either high doses of up to 160 mg tid or more frequent administration of betahistine could be advantageous for patients. This is currently being evaluated in an ongoing clinical trial.

In conclusion, this study showed that betahistine in clinically effective doses increases blood flow in cochlear stria vascularis vessels in a dose-dependent sigmoid way and thus is recommended for the treatment of Menière’s disease.
